# Pathological and Therapeutic Approach to Endotoxin-Secreting Bacteria Involved in Periodontal Disease

**DOI:** 10.3390/toxins13080533

**Published:** 2021-07-29

**Authors:** Rosalia Marcano, M. Ángeles Rojo, Damián Cordoba-Diaz, Manuel Garrosa

**Affiliations:** 1 Department of Cell Biology, Histology and Pharmacology, Faculty of Medicine and INCYL, University of Valladolid, 47005 Valladolid, Spain; rosaliamarcano@gmail.com; 2 Area of Experimental Sciences, Miguel de Cervantes European University, 47012 Valladolid, Spain; marojo@uemc.es; 3 Area of Pharmaceutics and Food Technology, Faculty of Pharmacy, and IUFI, Complutense University of Madrid, 28040 Madrid, Spain; damianco@farm.ucm.es

**Keywords:** endotoxins, LPS, lipopolysaccharide, *Porphyromonas gingivalis*, periodontal disease, fluoride, therapeutic approach

## Abstract

It is widely recognized that periodontal disease is an inflammatory entity of infectious origin, in which the immune activation of the host leads to the destruction of the supporting tissues of the tooth. Periodontal pathogenic bacteria like *Porphyromonas gingivalis*, that belongs to the complex net of oral microflora, exhibits a toxicogenic potential by releasing endotoxins, which are the lipopolysaccharide component (LPS) available in the outer cell wall of Gram-negative bacteria. Endotoxins are released into the tissues causing damage after the cell is lysed. There are three well-defined regions in the LPS: one of them, the lipid A, has a lipidic nature, and the other two, the Core and the O-antigen, have a glycosidic nature, all of them with independent and synergistic functions. Lipid A is the “bioactive center” of LPS, responsible for its toxicity, and shows great variability along bacteria. In general, endotoxins have specific receptors at the cells, causing a wide immunoinflammatory response by inducing the release of pro-inflammatory cytokines and the production of matrix metalloproteinases. This response is not coordinated, favoring the dissemination of LPS through blood vessels, as well as binding mainly to Toll-like receptor 4 (TLR4) expressed in the host cells, leading to the destruction of the tissues and the detrimental effect in some systemic pathologies. Lipid A can also act as a TLRs antagonist eliciting immune deregulation. Although bacterial endotoxins have been extensively studied clinically and in a laboratory, their effects on the oral cavity and particularly on periodontium deserve special attention since they affect the connective tissue that supports the tooth, and can be linked to advanced medical conditions. This review addresses the distribution of endotoxins associated with periodontal pathogenic bacteria and its relationship with systemic diseases, as well as the effect of some therapeutic alternatives.

## 1. Introduction

The oral cavity is one of the areas of living organisms where the highest rates of microorganisms are located. Among them, bacteria are the most common [1], and Gram-negative bacteria play a key role in oral infections. The virulence factors used by some of the bacteria involved in the evolution of oral infections include the release of lipopolysaccharide, a structural component of the bacterial cell wall that interacts with cells of host oral cavity connective tissue, modulating its immune response and able to cause diseases [2].

One of the oral infections that arouses greater interest due to its epidemiology is periodontal disease and its forms, periodontitis and gingivitis. Periodontitis is characterized by inflammation and destruction of connective and periradicular tissues, resulting from the interaction between microbial factors and the host immune response, which can lead to tooth loss [3]. Literature supports the link between periodontitis and systemic diseases [4], due to a continuous inflammation, bacterial circulation and bacterial products [5].

Among the periodontal pathogens, *P. gingivalis* is one of the most studied, capable of releasing large amounts of external vesicles containing endotoxins [6]. It is a Gram-negative bacterium, present in patients with periodontal disease (PD) that belongs to the group of black-pigmented Bacteroides and is often presented in the form of a coccobacterium. It can produce collagenase, proteases, hemolysins, endotoxins, fatty acids, ammonia, hydrogen sulfide and indole, among other products [5].

*P. gingivalis* is a late colonizer of the biofilm that forms after tooth brushing and the development of the glycoprotein of dental enamel [5]. It can penetrate the periodontal tissue and thus participate in the host destructive innate response associated with the disease [7]. The essential nutrients for the growth of *P. gingivalis* include hemin and phosphate. Given the impossibility of bacteria to retain iron [8], it makes them dependent on the heme group of erythrocytes, resulting in a decrease of oxygen in the periodontal tissue that favors the appearance of ischemia [9].

The pathogenic potential of *P. gingivalis* is not limited to the oral cavity; it can cause endotoxemia. For instance, *P. gingivalis* has been shown to influence glucose/lipid metabolism, hepatic steatosis, and the intestinal microbiota in mice [10]. Likewise, it alters cardiac function in mice by activating myocardial fibroblast cells [11]. Furthermore, in animal model studies it has been observed a close relation to rheumatoid arthritis [12,13]. An increasing number of studies supports the presence of *P*. *gingivalis*-LPS in brain tissue of individuals with Alzheimer’s disease 12-h postmortem [14]. These LPS also seem to promote atherogenesis and a serum lipid distribution eventually leading to vascular inflammation and lipid accumulation in macrophages [15].

Previous articles have checked the description of LPS biosynthesis [2,16,17,18] or the biological response of the endotoxin lipid A in the host cells [19]. Our review focuses on the periodontal disease and endotoxin-secreting bacteria involved in periodontitis, the endotoxin effect in oral tissues, their relationship with systemic diseases and the advances in therapeutic alternatives with emphasis in LPS control. Special attention is taken from a microscopic point of view to the possible biological markers, in order to avoid the potential damage to periodontal connective tissue supporting the teeth and the severe consequences in patients due to the distribution of endotoxins throughout the circulation.

## 2. Endotoxin as a Component of Gram-Negative Bacteria

An endotoxin is a LPS released by most Gram-negative bacteria and is located in the outer membrane of their cell wall [17,20,21,22,23]. The outer layer of the cell wall is composed of an asymmetric phospholipid bilayer that contains the LPS, and the inner layer of the membrane includes glycerophospholipids. LPS presence increases bacteria resistance to antimicrobial components and environmental stress [24,25].

**Figure 1:**
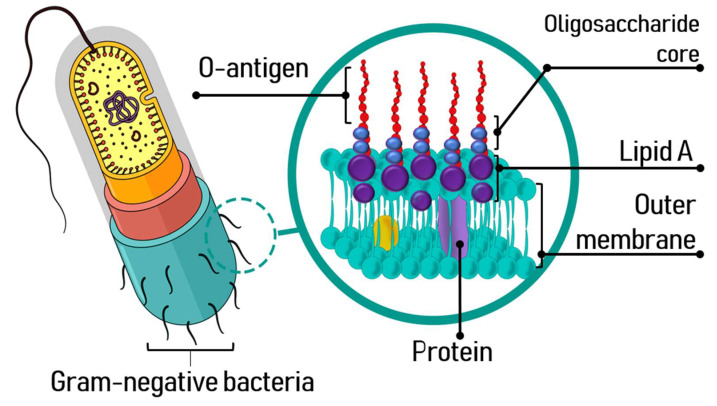
Endotoxin of Gram-negative bacteria.

The structure and composition of LPS, an amphipathic molecule, allows to establish ionic interactions with the components of the outer cell membrane, favoring the packing of LPS and altering the fluidity of the membrane. It is well known that endotoxins have three structural domains: lipid A, with a hydrophobic character, the core oligosaccharide and a polysaccharide portion known as O-antigen [2,24] (Figure 1). Its biological activity depends on the lipid A, a well-preserved part of the LPS, essential for its attachment to the bacterial outer membrane.

Lipid A is a phosphorylated glucosamine disaccharide acylated with hydroxyl saturated fatty acids [24,26], responsible for the toxic effects of Gram-negative bacterial infections. Saturated fatty acids further 3-O-acylate the 3-hydroxyl groups of the fatty acids of lipid A [19,27]. The core oligosaccharide bonds directly to lipid A and contributes to the bacterial viability and stability of the outer membrane. This phosphorylated heterooligosaccharide is also well-preserved in the proximal area to the lipid A.

Even though the biosynthetic pathway and LPS export mechanisms are common to most Gram-negative bacteria, the detailed structure of LPS varies from one bacterium to another and this could affect the virulence. Moreover, some pathogens can modify the basic structure of their LPS during the infection [17,19]. The difference between the LPS of several Gram-negative bacteria is in the length of the fatty acid chains and these seem to be related to the pathogenicity of the bacteria [28,29]. These variations are the basis of altered host immune response [16].

The O-antigen is a polysaccharide portion with a hydrophilic character and a major component with high variability on the surface of the LPS. Beside acting as a defense barrier in the bacterial cell, it facilitates its adhesion to host cells mediated by adhesins through the route of vesicular uptake [30]. The O-polysaccharide is formed of several units of oligosaccharide, and it can be homopolymeric or heteropolymeric. The LPS O-antigen confers antigenicity to the bacterial cell and thanks to the variability in its length, may avoid the control of the host immune system and escape from death [25,31,32,33]. The size and composition of the O-antigen is related to the virulence potential of the bacterial strain; hence, they play a fundamental role in the infection process, being a key factor for the interaction and colonization of the host cells as well as for the ability to bypass the defense mechanisms of the host [24].

In general, endotoxins are released by secretion, in vesicles formed on the bacterial outer membrane during the bacterium growth phase or are released during cell death, damaging periodontal tissues and triggering inflammation [34]. The vesicles can deliver virulence factors and modulate the host immune system during bacterial pathogenesis. LPS also are released when the cell is chemically treated to remove this glycolipid.

The LPS of *P. gingivalis* provides integrity to the bacterium and offers a mechanism for its interaction with other surfaces, allowing for the formation of biofilms [35]. During its growing phase, pathogenicity factors are released from the outer membrane vesicles (spherical microstructural bodies) [36,37], which are powerful stimulators of the innate immune signal transduction pathways in a tissue/cell-specific manner [38]. The *P. gingivalis*-LPS basic chemical composition is typical of a bacterial endotoxin with a main difference: the Lipid A structure can undergo isomeric acylation in two ways, tetraacylation and pentaacylation, depending on environmental factors such as hemin levels, phosphate availability and incubation temperatures; thus, eliciting differential immunoinflammatory responses [39].

There are two isolated forms of LPS from *P. gingivalis*: O-LPS and A-LPS. The main constitutive variation is the nature of their polysaccharide. O-LPS is a polysaccharide of the O-antigen tetrasaccharide repeating units, found in most Gram-negative bacteria, while A-LPS is an anionic polysaccharide repeating unit. Both, O- and A-LPS, are bound to Lipid A [9,39,40]. Furthermore, within the A-LPS, the nonphosphorylated penta-acylated and the nonphosphorylated tetra-acylated forms have been isolated. These lipopolysaccharides differ in size and are recognized by their molecular weight: LPS 1435/1449 for the tetra-acylated form and LPS 1690 for the penta-acylated form [41]. These regions follow different signaling pathways in the effector cells present in different organs and therefore, seems to be involved in different systemic diseases [9].

O-LPS and A-LPS play a key role in the pathogenic activity of *P. gingivalis*. Studies have shown the capacity of the nonpigmented mutant of *P. gingivalis* (mutant gtfB) with defects in the polysaccharide portions of O-LPS and A-LPS and its relation to a complete loss of gingipain-adhesion complexes, favoring auto-aggregation and an increased biofilm formation [42].

Some studies based on specific strains also observed a third form, the K-LPS, which contributes to the pathogenic effect of *P. gingivalis* by helping to maintain the structural integrity of the bacteria in hostile environments. Relative contributions of these LPS to the inflammatory potential of *P. gingivalis* and the possible variations in their proportions may influence the pathogenic phenotype [43].

Of the three components of LPS, the glycan part is responsible for the immunogenicity and can be used to detect the presence of an infection as it induces an innate immune response through Toll-like receptors (TLR). LPS constitutes the main antigenic surface of *P. gingivalis* and exhibits great activity in the human receptors TLR4 / MD2 / CD14 regarding to what is observed in the mouse [44]. The LPS is recognized by the complex TLR4/MD2, mediated by CD14 and accessory protein LBP, which induces the activation of several transcriptional regulators like factor nuclear kB (NF-kB), activator protein 1 (AP-1) and interferon (IFN) regulatory factors, leading to the expression of genes involved in the host immune response [45,46].

## 3. *Porphyromonas gingivalis* and Dental Biofilm

Dental biofilm is a complex system of multiple bacterial strains that cooperate and at the same time compete to colonize dental and periodontal tissues. Early interactions occur between oral surfaces and bacterial cells, enabling the conditions to a coaggregation process [47]. There are more than 700 bacterial species that can colonize the oral cavity, and a few have been identified as the main responsible for the expression of dental and periodontal disease [48,49,50]. The couple *Streptococcus oralis* and *Streptococcus mitis* on one hand; *Streptococcus gordonii* and *Streptococcus oralis* on the other hand; and finally, *Streptococcus sanguis* are identified as primary colonizers. Subsequently, a complex process of bacterial aggregation occurs with the incorporation of *Fusobacterium nucleatum* which guarantees, directly or through *Treponema denticola*, the adhesion of *P. gingivalis*. *P. gingivalis* is a late colonizer, like *Actinomyces actinomycetemcomitans* or the intermediate *Prevotella* [5,51]. Remarkably, most of the periodontopathogens are Gram-negative strictly anaerobic species [47] that require hemin and vitamin K to growth [52].

*P. gingivalis* can locally invade the periodontal tissue initiating immune and inflammatory responses [53,54]. It colonizes plaque biofilms at and below the gingival margin as well as other locations like the deep crypts of the tongue. The bacterial load is controlled by the host immune response, keeping the numbers low at the sulcus. However, changes in oral hygiene habits or in host responses can lead to an insufficient management and development of gingival inflammation, epithelial and connective tissue migration and compromised attachment between tooth and alveolar bone [55].

Bostanci et al. (2012) [49] describe *P. gingivalis* as a black-pigmented, assaccharolytic, non-motile Gram-negative microorganism that synthetizes amino acids to gain energy and needs anaerobic conditions to live. It penetrates gingival epithelial cells and pass through the epithelial barrier into deeper tissues. From the intracellular position, it uses the cellular recycling pathways to exit invaded cells and interfere with clot formation by digesting the fibrinogen, essential for wound healing, resulting in a persistent infection of periodontal tissues [56,57]. The microorganism adheres to the host cell surface in a process that follows the incorporation via lipid rafts, and the integration of the bacteria into early phagosomes, activating cellular autophagy and suppressing the apoptosis providing a replicative niche. The replicating vacuole contains host proteins delivered by autophagy that are used by *P. gingivalis* to survive and replicate within the host cell [58].

*P. gingivalis* can produce several virulence factors, which allow to evade host defense system and eventually cause damage and progression of periodontal disease, such as gingipains, collagenase, lectins, protease, superoxide dismutase (SOD) and LPS [7,38]. Furthermore, these products may enter the bloodstream through inflamed periodontal tissue and lymph vessels or via saliva to the gastrointestinal tract, from occasional procedures like dental practice or from daily routines like tooth brushing, leading to bacteremia or endotoxemia [59].

## 4. Immunoinflammatory Response in Oral Tissues Due to the Presence of Endotoxins Released by *P. gingivalis.* General Effects

**Figure 2:**
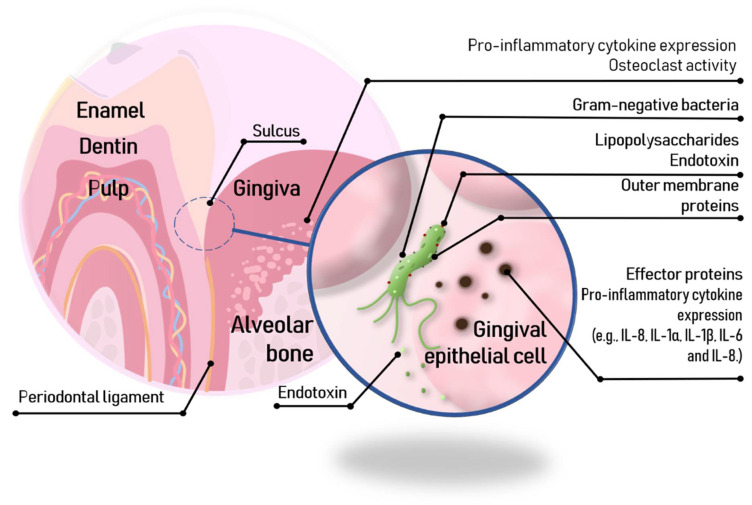
LPS adhesion to a gingival epithelial cell inducing the secretion of cytokines.

Inflammation is an immune response to an infectious agent and/or molecular hazard signal. Many innate immunity receptors participate in the inflammatory response and induce transcriptional activation to produce numerous cytokines, chemokines and other inflammatory mediators that can lead to osteoclastogenesis via osteoblast-related activities [60] (Figure 2). Cytokines of the IL-1 family, in addition to transcriptional activation, require proteolytic processing to generate cytokines with biological activity. This process is mediated by caspase-1, which in turn is controlled by several cytosolic multimolecular complexes, among them, the NLRP3 [49,61]. Chen et al. (2017) [62] observed by immunohistochemical studies in animal models that *P. gingivalis*-LPS is negatively regulated by NLRP3, precursor of IL-1_β_ (pro-IL-1_β_), and that IL-1_β_ matures under normoxia. Instead, under moderate hypoxia conditions (2% O_2_), *P. gingivalis*-LPS increased the expression of NLRP3 boosting the transcriptional activity of NF-kB.

**Figure 3:**
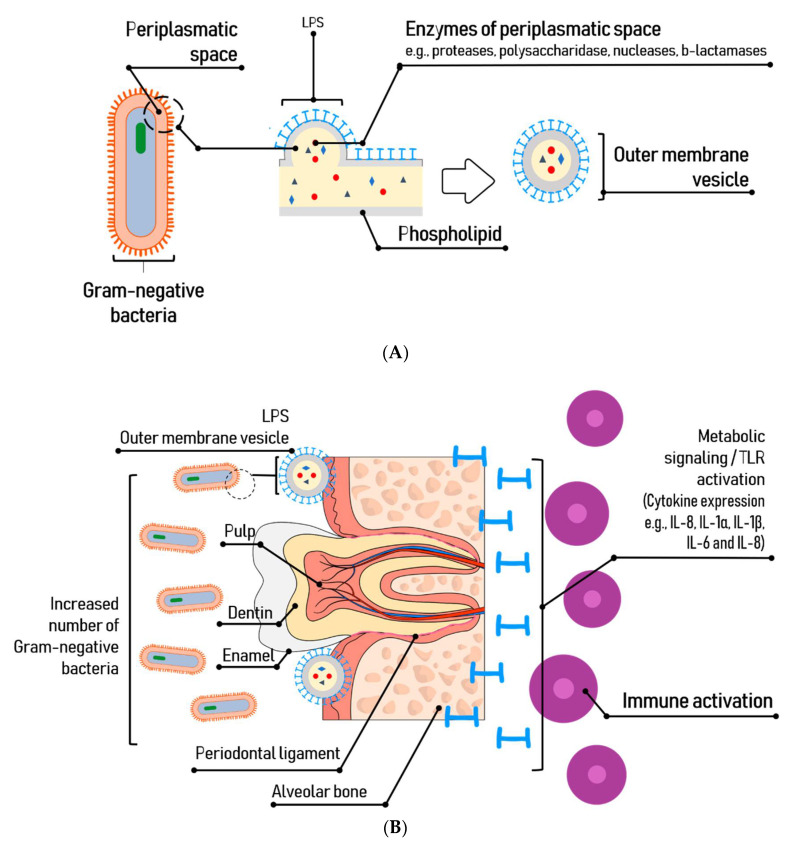
Release of LPS and Immune responses to *P. gingivalis*. (**A**): In the process of releasing LPS by budding, they accumulate as vesicles located on the bacterial outer membrane. (**B**): During the infection with *P. gingivalis*, its lipopolysaccharide stimulates the immune system located in the subjacent connective tissue by binding to TLR in the target cell. The LPS activates the TLR4 signaling pathway in recruited neutrophils, causing strong inflammatory responses designed to inactivate the pathogen.

In Gram-negative bacteria, lipid A activates responses by binding to TLR4 of the host innate immune system, which triggers the production of pro-inflammatory cytokines and promotes the elimination of bacteria (Figure 3). In the case of *P. gingivalis*, the lipid A is a TLR2 activator, and regarding TLR4, it has been observed that the penta-acylated form activates it, while the tetra-acylated form induces a weak antagonist activity on TLR4 and does not elicit a significant immuno-inflammatory effect [41]. Lipid A acylation depends on microenvironment conditions and *P. gingivalis* is able to modulate its binding to TLR receptors. Likewise, the A-LPS needed for cell integrity and serum resistance, is a weaker inducer of human monocyte cytokine responses, as compared to conventional LPS. The heterogenicity of *P. gingivalis*-LPS through its opposite actions favor immune deregulation. Strategically, this is in line with the manipulation of the host’s innate immune system. [49,63]. Moreover, we should highlight the phenomenon known as “tolerance to endotoxins”, consisting in a continuous exposure to LPS that produces a decrease in cytokine levels in response [64].

Bone resorption effect mediated by TLR2 has been observed in vitro in animal models [9]. Several authors referred to a decrease of oxygen and signs of ischemia apparently associated to the lysis of erythrocytes, allowing *P. gingivalis* to obtain heme to be fed and to grow persistently in favorable conditions [9,65,66,67].

The heterogenous forms of lipid A from LPS of *P. gingivalis* might be a key factor to understand how the host defense signaling mechanisms are altered and therefore deregulated [68,69]. Herath et al. (2013) [41] mentioned that the NF-ĸB signaling pathway was activated in human gingival fibroblasts (HGFs) by at least one form of LPS. The authors also refer to human fibroblasts and the secretion of a different profile of the pro-inflammatory cytokine expression like IL-6 and IL-8 and how a particular form of *P. gingivalis* LPS significantly regulated the expression of IL-6 and IL-8 mRNA at the gene level in HGFs [9,41]. Bozkurt et al. (2021) [60] studied the impact of *P. gingivalis* LPS in HGFs, observing a suppression of cell proliferation and an increase of pro-inflammatory changes in HGFs. Their findings suggest that *P. gingivalis* LPS-induced changes of the phenotypic and inflammatory characteristics in HGF, could potentially be a fundamental pathogenic mechanism for tissue destruction, resulting in extracellular matrix destruction by the increase of collagenolytic enzymes such as MMPs. In a subsequent study, the authors treated animal cementoblast cells (OCCM-30) with *P. gingivalis*-LPS, observing a significant induction of MMP-1 and MMP-2 plus the MMP-3 expression, indicating an excessive breakdown of the periodontal connective tissue.

According to Rangarajan et al. (2008) [70], the A-LPS induces production of IL-1_α_, IL-1_β_, IL-6 and IL-8. Pro-inflammatory genes were significantly upregulated by some isoforms of LPS. HGFs matrix metalloproteinase MMP-3 and its protein were upregulated by a penta-acylated *P. gingivalis*-LPS [41]. Lu et al. (2009) [71] refers to the upregulation of human beta-defensins, hBD-1, hBD-2, and hBD-3 mRNAs in human epithelia by an isoform of *P. gingivalis*. Furthermore, according to Ding et al. (2017) [72], the different isoforms of LPS also can affect periodontal pathogenesis by disrupting pattern recognition receptors (PRRs) like LPS-binding protein (LBP), helping a pathogen such as *P. gingivalis* to escape from host defenses, leading to persistent signs of periodontal disease.

When considering the mechanisms of bacteria to induce the release of pro-inflammatory substances we must keep in mind that the interaction between bacteria and host cells results in the release of one or more cytokines, their production depending mainly on the nature of the bacteria and the host cells involved. LPS modulates cell behavior due to induction of cytokine synthesis. Hence, the LPS of *P. gingivalis* can activate the host inflammatory and defense responses [73]. LPS is received primarily by TLR4, which is expressed by immune cells and other cell types. Induced by LPS, TLR4 activates the pro-inflammatory transcription factor NF-κB that enters the nucleus and initiates pro-inflammatory gene transcription that encode the pro-inflammatory cytokines, IL-1_β_, IL-6 and IL-8, leading to periodontal tissue destruction [54]. It increases the expression of Ephrin type-B receptor 4 (EphB4) and inhibits the expression of the protein EphrinB2 [74]. It also inhibits alkaline phosphatase activity, collagen type 1 Alpha 1, and the osteocalcin production and mineralization in the periodontal ligament stem cells proliferation. It also produces IL-1_β_, IL-6 and IL-8 [7,75].

Several clinical studies have shown an increase in levels of cytokine IL-17 in the serum of patients with aggressive periodontitis and elevated levels of IL-17 related cytokines in tissues with periodontal disease [76,77,78]. By means of immunopathological trials, it has been observed a significant correlation between IL-17 expression and bone loss in periodontitis, mediated by the generation of pathogenic Th17 (Effector T-Cell differentiated) cells. A constant presence of Th17 has been shown to support chronicity of inflammation and mediate tissue destruction due to the activation of resident matrix cells such as fibroblasts and osteoclasts [79,80,81].

## 5. Systemic Effects Related to Endotoxins Secreted by Microorganisms Involved in Periodontitis

The colonization of endodontic or periodontal tissues by microorganisms in carious, periodontal, and traumatic lesions leads to persistent infections such as dental pulpitis, dental necrosis, periodontitis and endo-perio lesions, among others [82]. Local infections of the oral cavity have been related to systemic conditions. The microbiota favors the exposure to the lipopolysaccharides resulting in a condition of metabolic endotoxemia with signs of low-grade inflammation, insulin resistance and increased cardiovascular risk [59,83]. The toxicity driven by the bacteria products can cause cell and tissue damage by the stimulation and release of chemical mediators [84] that appear to be closely related to the virulence and progress of oral pathologies [85]. Moreover, it has been reported that they target the macrophages, fibroblasts and neutrophiles [84,86], activate tumor necrosis factor (TNF) [84,87], interleukins (IL-1, IL-5, IL-6, IL-8) [84,85,88] prostaglandins, alpha-interferon, factor XII of coagulation and the complement system. This results in an increase of vascular permeability, neutrophile and vascular chemotaxis and lysozyme and lymphokine release, eventually causing local inflammatory reaction and alveolar bone resorption [84].

Besides local effects, a relation between oral diseases and systemic conditions such as atherosclerosis, diabetes, preterm birth, rheumatoid arthritis, pancreatic cancer and Alzheimer’s disease (AD) has been observed. LPSs can induce the release of prostaglandins and cytokines with a potential systemic effect such as inflammatory responses, cardiovascular, respiratory, cognitive and other dysfunctions [12,54,89,90,91].

### 5.1. Hepatology

Fujita et al. (2018) [90] established the relationship between non-alcoholic fatty liver disease and *P. gingivalis*-LPS. This microorganism was cultivated in order to extract LPS, and once obtained and purified, was daily injected in the right palatine gingiva of rats, resulting in mild fatty liver. This relationship was also detected due to the presence of lipid deposition and focal necrosis with inflammatory cells [90,92,93]. These observations are consistent with those reported by Isogai et al. (1988) [94], in which 10 μg of *P. gingivalis*-LPS injected into the maxillary oral vestibular mucosa of rats induced inflammation and edema whereas the intravenous injection of 100 μg resulted in necrotic lesions with many thrombi in the liver.

### 5.2. Diabetology

Chronic exposure of the host to LPS has been associated with insulin resistance, weight gain, and low-grade inflammation in animal models studies. High-fat diets facilitates the absorption of LPS across the intestinal barrier, resulting in inflammation [59,95]. Manco et al. (2009) [96] suggested that LPS is a factor that can trigger obesity and type-2 diabetes (T2D) associated with high-fat diets. Epidemiological studies in humans supports the association between periodontitis and elevated body weight [59,97,98,99,100]. Authors have observed a correlation between the levels of, for example, TNF-α in gingival crevicular fluid and plasma with the body mass index [101,102], and the expression of hyperlipidemia when higher values of periodontal disease parameters are observed [59,103].

Mesia et al. (2016) [104] studied the inflammatory responses in patients with T2D using blood samples stimulated with ultrapure *P. gingivalis*-LPS and proceeded with the quantification of cytokines/chemokines in culture supernatants. Their results demonstrated higher unstimulated levels of interleukin 6 (IL-6), IL-1_β_, tumor necrosis factor α, interferon γ, IL-10, IL-8, macrophage inflammatory protein 1α (MIP1_α_) and 1_β_ (MIP1_β_) and higher stimulated levels of IL-6, IL-8, IL-10, MIP1_α_ and MIP1_β_ in T2D. Moreover, the LPS-induced levels of IL-6, IL-8, IL-10 and MIP1_α_ were strongly associated with the severity of disease.

### 5.3. Neurology

Concerning the relation between *P. gingivalis*-LPS and cognitive mechanism, Zhang et al. (2018) [91] focused on the behavior and emotional changes in animals, spatial learning and memory, the activation of the microglia and astrocytes in cortex and hippocampus, the expression of cytokines and the activation of the TRL4 signaling pathway. Their results showed signs of memory loss. *P. gingivalis*-LPS plays an important role in the neurodegeneration and inflammation observed in patients with AD through recognition receptors (PRRs), such as TLRs, that stimulate CD14, TLR2 or TLR4 and send signals to the nucleus by the MyD88 (the adaptor for inflammatory signaling) pathway, triggering a cascade of events that result in an increased expression of proinflammatory cytokines.

Poole et al. (2013) [105] evaluated the presence of *P. gingivalis* and bacterial components using immunolabeling and immunoblotting in brain tissue of individuals with and without dementia. Their results showed the presence of *P. gingivalis*-LPS in AD cases, confirming that LPS from periodontal bacteria can reach the brain during life and could potentially contribute to the risk of progression of the disease.

Kamer et al. (2008) [106] suggested that periodontal disease can stimulate the production of amyloid beta (Aβ), the main component of the amyloid plaques found in the brains of people with AD and tau protein in the brain, eventually leading to the neuropathology. Wu et al. (2014) [14] observed that chronic systemic treatment with *P. gingivalis*-LPS induced the intracellular accumulation in the hippocampal pyramidal neurons of Aβ_1–42_ and chromo-granin A (CGA), a neurosecretory acidic glycoprotein present in senile plaques of patients with AD. This resulted in memory deficits in middle-aged mice.

Recent studies conclude that *P. gingivalis*-LPS in microglial cells could activate TLR2/TRL4-mediated NF-κB/STAT3 signaling pathways, leading to an immune-inflammatory response in BV-2 microglia cell line [107]. The continuous brain exposure to *P. gingivalis*-LPS initiated sarcopenia and cardiac injury without improving cognitive impairment [108,109].

### 5.4. Oncology

Regarding malignant pathologies, recent in vitro studies suggest a relation between *P. gingivalis*-LPS stimulation and the exacerbated production of pro-inflammatory cytokines in chronical affections like oral lichen planus (OLP), a precancerous condition that affects the oral mucosal stratified squamous epithelium and the underlying lamina propria. According to literature, the pathogenesis of OLP is associated with dysregulated T-cell responses to exogenous triggers and an antigen-specific mechanism by keratinocytes and Langerhans cells resulting in the activation of T-cells [110]. It has been observed that the normal buccal fibroblasts secreted weaker cytokines than the OLP associated fibroblast under LPS stimulation [110,111,112].

Both TLR2 and TLR4 are found frequently overexpressed in pancreatic ductal carcinoma. These TLRs recognize *P. gingivalis*-LPS. Lanki et al. (2018) [113] explored the effect of systemic administration of *P. gingivalis*-LPS in mouse pancreas. The LPS was prepared in physiological saline and administered intraperitoneally in a concentration of 5 mg/kg every 3 days for 1 month. DNA microarray analysis of gene expression, staining with hematoxylin–eosin and immunohistochemistry with anti-regenerating islet-derived 3A and G (*Reg3A/G*) antibody was performed. Data allowed to observe that *Reg3G*, a gene related to pancreatic cancer, was one of the 10 genes with the highest levels of expression in the pancreas stimulated with *P. gingivalis*-LPS suggesting the notion that periodontal disease may be a risk factor for pancreatic cancer. 

There is experimental evidence both in vitro and in vivo supporting that infection by *P. gingivalis* promotes distant metastases of oral cancer, as well as its resistance to anticancer agents. Woo et al. (2017) [114] have suggested that inflammatory signals are one of the most important factors to modulate chemoresistance and establish metastatic lesions, as was proved in tumor xenografts containing oral squamous cell carcinoma (OSCC) cells infected with *P. gingivalis* which showed greater resistance to paclitaxel (Taxol^®^) by activation of the Notch1 gene, as compared with tumor elicited with uninfected cells. The presence of a greater number of metastatic foci in the lung is even observed. These results led Woo et al. (2017) [114] to suggest that eradication of chronic periodontitis might serve as a therapeutic target for chemoresistant oral cancers, metastatic to the lung.

### 5.5. Rheumatology

Rheumatoid arthritis (RA) appears quite solidly related with periodontal disease. This chronic, inflammatory synovitis based on systemic immune disease, is mainly manifested as peripheral polyarthritis. It can cause the destruction of articular cartilage and joint capsule and provoke joint deformities [115,116,117]. *P. gingivalis*-LPS, along with fimbriae and gingipains, ensure the activation of TLR2, TLR4, nucleotide-binding oligomerization domain-containing protein 2 (NOD2) and proteinase-activated receptor 2 (PAR2), leading to inflammation. TLRs have been implicated in triggering and perpetuation of synovial events with the expression of TLRS 2, 3, 4, 6, 7 and 9 demonstrated in the rheumatoid joint. Bacterial endotoxins bound to TLR- receptors of the host oral cells, contributes to pathogenicity and triggers periodontal disease [118]. Plaque accumulation and maturation over time plus the lack of hygiene countermeasures, as well as susceptibility of the host will lead to composition changes in the biofilms, allowing for the proliferation of Gram-negative bacteria [119]. These pathogens exhibit metabolic features determinant for their virulence such as the production of proteases, hydrogen sulfides, fatty acids and the molecular properties of the LPS component of their wall [120,121,122,123]. Furthermore, dysregulation of the cytokine network and aberrant activation of leukocytes participating in the innate immune response against periodontal pathogens, activate the complement system, the receptor activator for NF-κB ligand and the signaling pathways, as well as the differentiation of T-helper cells, which contribute to the activation of the osteoclasts in affected joints [12].

## 6. Polymicrobial Oral Synergy and Endotoxins

The presence of periodontal pathogens and their metabolic subproducts in the mouth modulate the immune response beyond the oral cavity, thus promoting the development of systemic pathologies. The most frequent microorganisms in periodontal diseases are *Treponema, Bacteroides, Porphyromonas, Prevotella, Capnocytophaga, Peptostreptococcus, Fusobacterium, Actinobacillus* and *Eikenella* [124].

Bacteria from the *Actinomyces* genus are common for the oral microorganism flora. These microorganisms have an important role in the biofilm formation and plaque accumulation in both supra and subgingival locations [125]. These optional anaerobic Gram-positive bacteria are very often associated with caries, primary and secondary endodontic infection, as well as with a progression of tooth decay [126]. Evidence places these microorganisms among the main responsible of persistent extraradicular infections, complicated endodontic treatments and periapical lesions in the presence or not of root canal restorations, as was concluded by Dioguardi et al. (2020) [82], in a systematic review that provided data on the prevalence of the Actinomyces in persistent root lesions. It is also mentioned that infection can occur via mucosal lesions, endodontic pathways and periodontal disease.

Könönen et al. (2015) [127] refers to the clinical expression of human actinomycosis as an indolent, slowly progressing granulomatous disease, which can be categorized according to the body site, as orocervicofacial, thoracic and abdominopelvic forms. Other forms include cutaneous actinomycosis, pericarditis, infection of the nervous central system, macroglossia and osteonecrosis of the jaw associated with bisphosphonate treatment, as well as disseminated disease. Signs and symptoms include a persistent mass lesion accompanied with abscess formation, fibrosis, and sulfur granules, swelling, cough, low-grade fever and weight loss.

*Fusobacterium nucleatum*, an anaerobic Gram-negative bacterium, located in the intermediate layers of mature subgingival biofilms [128], is associated with periodontitis and colorectal cancer [129,130]. Histological studies have revealed that macrophages induced by *F. nucleatum* aggravate the progression of colitis [131] and are also related with complications of pregnancy [132], the synthesis of LPS being important to be considered among its pathogenicity factors [133]. Furthermore, it has been observed that *F. nucleatum* shows a synergy with the presence of *P. gingivalis* favoring the loss of alveolar bone [134]. Both microorganisms increased the gene expression of TLR2 and TLR4 [135,136]. However, when analyzing the relationship between periodontitis and arthritis in mice, in which a mixture of *P. gingivalis*, *F. nucleatum* and *Actinobacillus actinomycetemcomintans* was inoculated, less loss of alveolar bone was observed than when mice were inoculated with *P. gingivalis* alone, and oral inoculation with *F. nucleatum* or *A. actinomycetemcomintans* alone accelerated the subsequent onset and progression of arthritis [137].

*A. actinomycetemcomitans*, a periodontal bacterium [138], contains multiple virulence factors, including lipopolysaccharides, that can activate host inflammatory responses to initiate alveolar bone resorption [139,140]. Like *P. gingivalis*, it produces cytokines in reaction to the inflammatory response. *A. actinomycetemcomitans* and *P. gingivalis* have been associated with coronary heart disease and by means of immunofluorescent microscopy tests, it has been proved that they also were able to invade cells of the atherosclerotic plaque [141].

The latest review by Aquino-Martinez (2021) [142] highlights how the dissemination of periodontal bacteria into lung tissues may cause lipopolysaccharide-induced senescence, which facilitates SARS-CoV-2 cell attachment, entry and replication. The authors explain that LPS interacts with the SARS-CoV-2 spike protein, which potentiates the NF-kB activation, and referred to recent publications where a connection between periodontitis and COVID-19 is suggested by a common proinflammatory cytokine expression profile. Patients with severe symptoms of COVID-19 have increased IL-1, IL-7, IL-10, IL-17, IL-8, TNF and MCP-1 serum levels when also afflicted with periodontal disease. Some of these cytokines appear to have an important role in exacerbating lung condition and even can serve as a biomarker of COVID-19.

Patients with SARS-CoV-2 might present progressive respiratory failure, the most common cause to require intensive care assistance. The intubation and invasive ventilation necessary in some of the cases is a high-risk procedure with potential complications as the ventilator-associated pneumonia (VAP) described by Aquino-Martinez et al. (2021) [142]. The disease is a polymicrobial infection, but the major group of pathogens isolated from samples of patients with VAP is Gram-negative bacteria like periodontal pathogens and bacteria from the dorsal surface of the tongue or those contaminated present in upper air-way secretions. This can be directly implicated in the pathogenesis of VAP and can possibly lead to an increased secretion of cytokines.

## 7. Therapeutic Approaches of Oral Diseases and Their Effect on LPS

### 7.1. Fluoride as Bacterial LPS Inactivator

In general, preventive and therapeutic approach of periodontal and other oral diseases, includes plaque control to reduce microorganism aggregation. This is achieved by sustained and frequent oral hygiene employing mechanical and chemical tools to lower the viral burden. Fluoride has been widely used in dentistry due to its anticaries effect, antimicrobial properties, and desensitizing potential [143,144,145,146,147,148,149].

Madléna et al. (2012) [150] observed a decrease in plaque index, gingival index and bleeding when testing in orthodontic patients using amine fluoride and stannous fluoride (Am/SnF_2_) toothpaste in combination or not with a mouth rinse with Am/SnF_2_. Under these conditions, the dental plaque shifts towards less acidogenic, thus supporting an antibacterial property of fluoride agents [150,151].

Haught et al. (2016) [152] applied antimicrobial solutions, containing stannous fluoride (SnF_2_), to LPS from *E. coli* and *P. gingivalis* to determine, by fluorescence assays and mass spectroscopy, the binding ability of SnF_2_. Stannous fluoride interfered with LPS and inhibited the binding to TLR4 in both dying and cellular assays, hence, potentially reducing their effect in the host cells. In another study, stannous fluoride inhibited gene expression response of TLR4 and TLR2 in HEK293 cells, producing a complete inhibition at micromolar concentrations. Moreover, the addition of stannous fluoride suppressed production of TNF_α_, IFN-g, IL-12p70, IL10, IL-1b, IL-2 and IL-6, and increased secretion of IL-8. Thus, stannous fluoride had the potential to provide benefits in the early signs of periodontal disease, directly decreasing the pathogenicity of plaque biofilms by blocking reactivity of LPS with tissue receptors associated with inflammation [153].

Clinical improvements in gingivitis also have been reported after applying stannous fluoride dentifrice [118]. These authors found significant changes in the number of cultivable Gram-negative organisms in sampled supragingival and subgingival plaque and a considerable reduction in promotion of TLR activation for subgingival plaque samples. Furthermore, Xie et al. (2018) [154], in an expanded analysis of previous studies, observed the hygienic treatment effects of stabilized stannous fluoride tooth paste on chemically measured endotoxins and the activation of TLR based gene expression in TLR2 specific cell line and a THP-1 (multi TLR reporter) cell line. These authors found that SnF_2_ dentifrice treatment potentially reduced the endotoxin content and virulence potentiation properties of subgingival dental plaque, therefore concluding that SnF_2_ might be beneficial to reduce the pathogenicity of subgingival dental plaque.

### 7.2. Surgical and Non-Surgical Periodontal Treatment as a LPS Modulator

Authors agree that periodontal treatment can reduce the bacterial burden and therefore the level of LPS, attenuating their potential inflammatory effect in short term. The therapeutic approach, consisting in professional plaque removal together with home-care reinforcement, combined with an antibiotic treatment based in 500 mg amoxicillin and 250 mg metronidazole, three times a day for seven days, was effective in partially modulating LPS responsiveness [155]. 

Following the periodontal treatment protocol for patients with localized aggressive periodontitis (LAP) described by Shaddox et al. (2013) [155], in which an ultrasonic full-mouth debridement, site-specific scaling and root planning was performed together with the prescription of wide spectrum antibiotics, combined with home-care instructions, a decrease in clinical parameters of the disease was observed probably due to a reduction in the cytokine/chemokine LPS response after treatment [156]. These studies refer to a rebound effect in LPS inflammatory response after six to twelve months, that apparently do not influence the ability of high responsive patients to show reductions in their clinical parameters of disease, likely due to LPS tolerance in these subjects [155,156,157].

### 7.3. LPS Neutralization by High Density Lipoproteins

Most of lipoproteins have been reported to bind LPS. The high-density lipoproteins (HDLs) seem to be the more efficient for binding and inactivating different types of LPS. According to Meilhac (2012) [158], the plasma phospholipid-transfer protein (PLTP) was then reported to transfer LPS to HDL in conjunction with the LPS-binding protein (LBP) leading to LPS neutralization. LBP and PLTP can remove LPS from bacterial membranes and transfer it to HDLs [158,159]. Lipid A diglucosamine-phosphate region seems to be responsible for the association of LPS with HDLs and its neutralization relies on LBP, which may form a complex between CD14 and LPS, favoring its binding to HDL particles and subsequent neutralization, revealing a protective and possible contributory potential in periodontal disease treatment [158,160].

## 8. Concluding Remarks

From an epidemiologic point of view, periodontal disease is one of the most worrying of oral diseases. Through an immunologic activation directed by endotoxins of Gram-negative bacteria present in the dental biofilms, it can lead to the destruction of the supporting tissues of the teeth. In addition, numerous experimental studies offer sufficient evidence that periodontitis adversely impacts systemic health through biologically plausible mechanisms.

More than 700 bacterial species colonize the oral cavity, Gram-negative strictly anaerobes, such as *P. gingivalis,* being identified as main periodontopathogens. The release of LPS present in the outer cell wall of these bacteria either in vesicles or after bacterial lysis, causes tissue damage and modulation of the immune response in the host. Among components of LPS, lipid A is responsible for the toxic effects, while O-antigen is a key factor for the interaction and colonization of host cell.

Changes in oral hygiene habits and/or in host responses, together with bacterial virulence factors like gingipains, collagenase, lectins, proteases, SOD and LPS, lead to occurrence of pathologies. In this regard, endotoxins secreted in oral cavity infections cause not only oral pathological changes, such as gingival inflammation, epithelial and connective tissue migration or compromised attachment between tooth and alveolar bone, but also endotoxemia leading to systemic deleterious repercussions on the outcome of clinical entities such as atherosclerosis, diabetes, preterm birth, non-alcoholic fatty liver, arthritis, colorectal cancer, oral cancer metastases, coronary heart disease, Alzheimer’s disease and COVID-19.

Further consideration to highlight is that endotoxins, by binding to Toll-like receptors 4 (TLR4) in the innate immune system of the host, activates nuclear factor κB (NFκB) triggering the production of pro-inflammatory cytokines to promote bacteria destruction. At the same time, bacteria defense themselves by means of LPS lipid A which may act as a TLR4 antagonist leading to immune deregulation and disrupting the pattern recognition receptors in host cells to help the pathogen escape from death. The composition of the microbiota present in the oral cavity also must be taken into account since synergistic actions among different bacteria have been described, such as the association between *Fusobacterium nucleatum* and *P.gingivalis* favoring loss of alveolar bone.

Given the importance of these infections and their release of endotoxins leading to the mentioned systemic repercussions, they deserve much greater attention. Therefore, more research is needed to find out the mechanisms whereby LPS provokes deregulation of the innate immune system and why particular systemic pathologies are influenced, in order to apply preventive actions, early detection and curative treatment. Currently, periodontitis, gingivitis and other oral infections have been proved to respond favorably to stannous fluoride or to a mixture of it with amine fluoride, albeit this response varies across the different bacterial species. Evidence supports that through a clinical periodontal treatment the serum level of inflammatory factors is decreased while metabolic control and other markers of systemic diseases are improved. Maintenance is also necessary, and therefore, the use of other therapeutic techniques and supportive measures like fluoride exposure are needed. Fluoride seems to decrease the number of microorganisms and blocks the binding of endotoxins to TLR, making it suitable as a component to be employed together with mechanical tools in a sustained and frequent oral hygiene for plaque control. The recommendation to modulate LPS in oral infections is a professional plaque removal along with home-care reinforcement combined with wide spectrum antibiotic treatment. In addition, neutralization of LPS with lipoprotein also appears as a potential therapeutic approach.

## Data Availability

Not applicable.

## References

[1] Siqueira J.F., Rôças I.N. (2009). Diversity of endodontic microbiota revisited. J. Dent. Res..

[2] Bertani B., Ruiz N. (2018). Function and biogenesis of lipopolysaccharides. EcoSal Plus.

[3] Pihlstrom B.L., Michalowicz B.S., Johnson N.P. (2005). Periodontal Diseases. Lancet.

[4] Bui F., Almeida-da-Silva C., Huynh B., Trinh A., Liu J., Woodward J., Asadi H., Ojcius D. (2019). Association between periodontal pathogens and systemic disease. Biomed. J..

[5] Fiorillo L., Cervino G., Laino L., D’Amico C., Mauceri R., Tozum T.F., Gaeta M., Cicciù M. (2019). *Porphyromonas gingivalis*, periodontal and systemic implications: A systematic review. Dent. J..

[6] Roier S., Zingl F.G., Cakar F. (2016). A novel mechanism for the biogenesis of outer membrane vesicles in Gram-negative bacteria. Nat. Commun..

[7] Mysak J., Podzimek S., Sommerova P., Lyuya-Mi Y., Bartova J., Janatova T., Prochazkova J., Duskova J. (2014). *Porphyromonas gingivalis*: Major periodontopathic pathogen overview. J. Immunol. Res..

[8] Rangarajan M., Aduse-Opoku J., Paramonov N. (2017). Hemin binding by *Porphyromonas gingivalis* strains is dependent on the presence of A-LPS. Mol. Oral Microbiol..

[9] Olsen I., Singhrao S.K. (2018). Importance of heterogeneity in *Porhyromonas gingivalis* lipopolysaccharide lipid A in tissue specific inflammatory signalling. J. Oral Microbiol..

[10] Sasaki N., Katagiri S., Komazaki R., Watanabe K., Maekawa S., Shiba T., Udagawa S., Takeuchi Y., Ohtsu A., Kohda T. (2018). Endotoxemia by *Porphyromonas gingivalis* injection aggravates non-alcoholic fatty liver disease, disrupts glucose/lipid metabolism, and alters gut microbiota in mice. Front. Microbiol..

[11] DeLeon-Pennell K.Y., Iyer R.P., Ero O.K., Cates C.A., Flynn E.R., Cannon P.L., Jung M., Shannon D., Garrett M.R., Buchanan W. (2017). Periodontal-induced chronic inflammation triggers macrophage secretion of Ccl12 to inhibit fibroblast-mediated cardiac wound healing. JCI Insight.

[12] Potempa J., Mydel P., Koziel J. (2017). The case for periodontitis in the pathogenesis of rheumatoid arthritis. Nat. Rev. Rheumatol..

[13] Perricone C., Ceccarelli F., Saccucci M., Di Carlo G., Bogdanos D.P., Lucchetti R., Pilloni A., Valesini G., Polimeni A., Conti F. (2019). *Porphyromonas gingivalis* and rheumatoid arthritis. Curr. Opin. Rheumatol..

[14] Wu Z., Nakanishi H. (2014). Connection between periodontitis and Alzheimer’s disease: Possible roles of microglia and leptomeningeal cells. J. Pharm. Sci..

[15] Chistiakov D.A., Orekhov A.N., Bobryshev Y.V. (2016). Links between atherosclerotic and periodontal disease. Exp. Mol. Pathol..

[16] Gronow S., Brade H. (2001). Lipopolysaccharide biosynthesis: Which steps do bacteria need to survive?. J. Endotoxin Res..

[17] Wang X., Quinn P.J. (2010). Endotoxins: Lipopolysaccharides of gram-negative bacteria. Subcell. Biochem..

[18] Klein G., Raina S. (2019). Regulated assembly of LPS, its structural alterations and cellular response to LPS defects. Int. J. Mol. Sci..

[19] Gao J., Guo Z. (2018). Progress in the synthesis and biological evaluation of lipid A and its derivatives. Med. Res. Rev..

[20] Silhavy T.J., Kahne D., Walker S. (2010). The bacterial cell envelope. Cold Spring Harb. Perspect. Biol..

[21] Rhee S.H. (2014). Lipopolysaccharide: Basic biochemistry, intracellular signaling, and physiological impacts in the gut. Intest. Res..

[22] Sampath V. (2018). Bacterial endotoxin-lipopolysaccharide; structure, function and its role in immunity in vertebrates and invertebrates. Agric. Nat. Res..

[23] Martyanov A.A., Maiorov A.S., Filkova A.A., Ryabykh A.A., Svidelskaya G.S., Artemenko E.O., Gambaryan S.P., Panteleev M.A., Sveshnikova A.N. (2020). Effects of bacterial lipopolysaccharides on platelet function: Inhibition of weak platelet activation. Sci. Rep..

[24] Raetz C.R., Whitfield C. (2002). Lipopolysaccharide endotoxins. Annu. Rev. Biochem..

[25] Silipo A., Molinaro A., Wang X., Quinn P. (2010). The diversity of the core oligosaccharide in lipopolysaccharides. Endotoxins: Structure, Function and Recognition. Subcellular Biochemistry.

[26] Dixon D.R., Darveau R.P. (2005). Lipopolysaccharide heterogeneity: Innate host responses to bacterial modification of lipid a structure. J. Dent. Res..

[27] Zamyatina A., Beilstein J. (2018). Aminosugar-based immunomodulator lipid A: Synthetic approaches. Org. Chem..

[28] Varbanets L.D. (2016). Structure, function and biological activity of lipopolysaccharide lipid A. Mikrobiol. Z..

[29] Steimle A., Autenrieth I.B., Frick J.S. (2016). Structure and function: Lipid A modifications in commensals and pathogens. Int. J. Med. Microbiol..

[30] O’Donoghue E.J., Sirisaengtaksin N., Browning D.F., Bielska E., Hadis M., Fernandez-Trillo F., Alder-wick L., Jabbari S., Krachler A.M. (2017). Lipopolysaccharide structure impacts the entry kinetics of bacterial outer membrane vesicles into host cells. PLoS Pathog..

[31] Rosenfeld Y., Shai Y. (2006). Lipopolysaccharide (Endotoxin)-host defense antibacterial peptides interactions: Role in bacterial resistance and prevention of sepsis. Biochim. Biophys. Acta.

[32] Matsuura M. (2013). Structural Modifications of bacterial lipopolysaccharide that facilitate gram-negative bacteria evasion of host innate immunity. Front. Immunol..

[33] Farhana A., Khan Y.S. (2020). Biochemistry, Lipopolysaccharide.

[34] Schwechheimer C., Kuehn M.J. (2015). Outer-membrane vesicles from Gram-negative bacteria: Biogenesis and functions. Nat. Rev. Microbiol..

[35] Li Y., Shi Z., Radauer-Preiml I., Andosch A., Casals E., Luetz-Meindl U., Cobaleda M., Lin Z., Jaberi-Douraki M., Italiani P. (2017). Bacterial endotoxin (lipopolysaccharide) binds to the surface of gold nanoparticles, interferes with biocorona formation and induces human monocyte inflammatory activation. Nanotoxicology.

[36] Veith P.D., Chen Y.Y., Gorasia D.G., Chen D., Glew M.D., O’Brien-Simpson N.M., Cecil J.D., Holden J.A., Reynolds E.C. (2014). *Porphyromonas gingivalis* outer membrane vesicles exclusively contain outer membrane and periplasmic proteins and carry a cargo enriched with virulence factors. J. Proteome Res..

[37] Xie H. (2015). Biogenesis and function of *Porphyromonas gingivalis* outer membrane vesicles. Future Microbiol..

[38] Jia L., Han N., Du J., Guo L., Luo Z., Liu Y. (2019). Pathogenesis of important virulence factors of *Porphyromonas gingivalis* via toll-like receptors. Front. Cell. Infect. Microbiol..

[39] Cutler C.W., Eke P.I., Genco C.A., Van Dyke T.E., Arnold R.R. (1996). Hemin-induced modifications of the antigenicity and hemin-binding capacity of *Porphyromonas gingivalis* lipopolysaccharide. Infect Immun..

[40] Paramonov N.A., Aduse-Opoku J., Hashim A., Rangarajan M., Curtis M.A. (2009). Structural analysis of the core region of O-lipopolysaccharide of *Porphyromonas gingivalis* from mutants defective in O-antigen ligase and O-antigen polymerase. J. Bacteriol..

[41] Herath T.D., Wang Y., Seneviratne C.J. (2013). The expression and regulation of matrix metalloproteinase-3 is critically modulated by *Porphyromonas gingivalis* lipopolysaccharide with heterogeneous lipid A structures in human gingival fibroblasts. BMC Microbiol..

[42] Yamaguchi M., Sato K., Yukitake H., Noiri Y., Ebisu S., Nakayama K. (2010). A *Porphyromonas gingivalis* mutant defective in a putative glycosyltransferase exhibits defective biosynthesis of the polysaccharide portions of lipopolysaccharide, decreased gingipain activities, strong autoaggregation, and increased biofilm formation. Infect. Immun..

[43] Aduse-Opoku J., Slaney J.M., Hashim A., Gallagher A., Gallagher R.P., Rangarajan M., Boutaga K., Laine M.L., Van Winkelhoff A.J., Curtis M.A. (2006). Identification and characterization of the capsular polysaccharide (K-antigen) locus of *Porphyromonas gingivalis*. Infect. Immun..

[44] Nativel B., Couret D., Giraud P., Meilhac O., d’Hellencourt C.L., Viranaïcken W., Da Silva C.R. (2017). *Porphyromonas gingivalis* lipopolysaccharides act exclusively through TLR4 with a resilience between mouse and human. Sci. Rep..

[45] Tan Y., Kagan J.C. (2014). A cross-disciplinary perspective on the innate immune responses to bacterial lipopolysaccharide. Mol. Cell..

[46] Kim S.J., Kim H.M. (2017). Dynamic lipopolysaccharide transfer cascade to TLR4/MD2 complex via LBP and CD14. BMB Rep..

[47] Hojo K., Nagaoka S., Ohshima T., Maeda N. (2009). Bacterial interactions in dental biofilm development. J. Dent. Res..

[48] Aas J.A., Paster B.J., Stokes L.N., Olsen I., Dewhirst F.E. (2005). Defining the normal bacterial flora of the oral cavity. J. Clin. Microbiol..

[49] Bostanci N., Belibasakis G.N. (2012). *Porphyromonas gingivalis*: An invasive and evasive opportunistic oral pathogen. FEMS Microbiol. Lett..

[50] Arora N., Mishra A., Chugh S. (2014). Microbial role in periodontitis: Have we reached the top? Some unsung bacteria other than red complex. J. Indian Soc. Periodontol..

[51] Lindhe J. (2009). Parodontologia Clinica E Implantologia Orale.

[52] Al-Qutub M.N., Braham P.H., Karimi-Naser L.M., Liu X., Genco C.A., Darveau R.P. (2006). Hemin-dependent modulation of the lipid A structure of *Porphyromonas gingivalis* lipopolysaccharide. Infect. Immun..

[53] Tribble G.D., Kerr J.E., Wang B.Y. (2013). Genetic diversity in the oral pathogen *Porphyromonas gingivalis*: Molecular mechanisms and biological consequences. Future Microbiol..

[54] Dahlen G., Basic A., Bylund J. (2019). Importance of virulence factors for the persistence of oral bacteria in the inflamed gingival crevice and in the pathogenesis of periodontal disease. J. Clin. Med..

[55] Armitage G.C. (2004). Periodontal diagnoses and classification of periodontal diseases. Periodontol 2000.

[56] Imamura T., Potempa J., Pike R.N., Moore J.N., Barton M.H., Travis J. (1995). Effect of free and vesicle-bound cysteine proteinases of *Porphyromonas gingivalis* on plasma clot formation: Implications for bleeding tendency at periodontitis sites. Infect. Immun..

[57] Sakanaka A., Takeuchi H., Kuboniwa M., Amano A. (2016). Dual lifestyle of *Porphyromonas gingivalis* in biofilm and gingival cells. Microb. Pathog..

[58] Bélanger M., Rodrigues P.H., Dunn W.A., Progulske-Fox A. (2006). Autophagy: A highway for *Porphyromonas gingivalis* in endothelial cells. Autophagy.

[59] Kallio E. (2014). Lipopolysaccharide: A Link Between Periodontitis and Cardiometabolic Disorders.

[60] Bozkurt S.B., Tuncer Gokdag I., Hakki S.S. (2021). *Porphyromonas gingivalis*-Lipopolysaccharide induces cytokines and enzymes of the mouse cementoblasts. Cytokine.

[61] Kajiwara K., Takata S., To T.T., Takara K., Hatakeyama Y., Tamaoki S., Darveau R.P., Ishikawa H., Sawa Y. (2017). The promotion of nephropathy by *Porphyromonas gingivalis* lipopolysaccharide via toll-like receptors. Diabetol. Metab. Syndr..

[62] Cheng R., Liu W., Zhang R., Feng Y., Bhowmick N.A., Hu T. (2017). *Porphyromonas gingivalis*-derived lipopolysaccharide combines hypoxia to induce caspase-1 activation in periodontitis. Front. Cell Infect. Microbiol..

[63] Hajishengallis G., Wang M., Liang S. (2009). Induction of distinct TLR2-mediated proinflammatory and proadhesive signaling pathways in response to *Porphyromonas gingivalis* fimbriae. J. Immunol..

[64] Brown J., Wang H., Hajishengallis G., Martin M. (2011). TLR-signaling Networks: An integration of adaptor molecules, kinases, and cross-talk. J. Dent. Res..

[65] Gibson F.C., Ukai T., Genco C.A. (2008). Engagement of specific innate immune signaling pathways during *Porphyromonas gingivalis* induced chronic inflammation and atherosclerosis. Front. Biosci..

[66] Ukai T., Yumoto H., Gibson F.C. (2008). Macrophage-elicited osteoclastogenesis in response to bacterial stimulation requires Toll-like receptor 2-dependent tumor necrosis factor-alpha production. Infect. Immun..

[67] Papadopoulos G., Weinberg E.O., Massari P. (2013). Macrophage-specific TLR2 signaling mediates pathogen-induced TNF-dependent inflammatory oral bone loss. J. Immunol..

[68] Trent M.S., Stead C.M., Tran A.X. (2006). Diversity of endotoxin and its impact on pathogenesis. J. Endotoxin Res..

[69] Coats S.R., Jones J.W., Do C.T. (2009). Human Toll-like receptor 4 responses to *P. gingivalis* are regulated by lipid A 1- and 4ʹ-phosphatase activities. Cell Microbiol..

[70] Rangarajan M., Aduse-Opoku J., Paramonov N. (2008). Identification of a second lipopolysaccharide in *Porphyromonas gingivalis* W50. J. Bacteriol..

[71] Lu Q., Darveau R.P., Samaranayake L.P., Wang C.Y., Jin L. (2009). Differential modulation of human {beta}-defensins expression in human gingival epithelia by *Porphyromonas gingivalis* lipopolysaccharide with tetra- and penta-acylated lipid A structures. Innate Immun..

[72] Ding P.H., Darveau R.P., Wang C.Y. (2017). 3LPS-binding protein and its interactions with *P. gingivalis* LPS modulate pro-inflammatory response and Toll-like receptor signaling in human oral keratinocytes. PLoS ONE.

[73] Zhang Y., Wang X.C., Bao X.F., Hu M., Yu W.X. (2014). Effects of *Porphyromonas gingivalis* lipopolysaccharide on osteoblast-osteoclast bidirectional EphB4-EphrinB2 signaling. Exp. Ther. Med..

[74] Zhu S., Liu Z., Yuan C., Lin Y., Yang Y., Wang H., Zhang C., Wang P., Gu M. (2020). Bidirectional ephrinB2 EphB4 signaling regulates the osteogenic differentiation of canine periodontal ligament stem cells. Int. J. Mol. Med..

[75] Kato H., Taguchi Y., Tominaga K., Umeda M., Tanaka A. (2014). *Porphyromonas gingivalis* LPS inhibits osteoblastic differentiation and promotes pro-inflammatory cytokine production in human periodontal ligament stem cells. Arch. Oral Biol..

[76] Cardoso C.R., Garlet G.P., Crippa G.E., Rosa A.L., Junior W.M., Rossi M.A. (2009). Evidence of the presence of T helper type 17 cells in chronic lesions of human periodontal disease. Oral Microbiol. Immunol..

[77] Nares S., Moutsopoulos N.M., Angelov N., Rangel Z.G., Munson P.J., Sinha N. (2009). Rapid myeloid cell transcriptional and proteomic responses to periodontopathogenic *Porphyromonas gingivalis*. Am. J. Pathol..

[78] Ohyama H., Kato-Kogoe N., Kuhara A., Nishimura F., Nakasho K., Yamanegi K. (2009). The involvement of IL-23 and the Th17 pathway in periodontitis. J. Dent. Res..

[79] Sato K., Suematsu A., Okamoto K., Yamaguchi A., Morishita Y., Kadono Y. (2006). Th17 functions as an osteoclastogenic helper T cell subset that links T cell activation and bone destruction. J. Exp. Med..

[80] Takayanagi H. (2009). Osteoimmunology and the effects of the immune system on bone. Nat. Rev. Rheumatol..

[81] Moutsopoulos N.M., Kling H.M., Angelov N. (2012). *Porphyromonas gingivalis* promotes Th17 inducing pathways in chronic periodontitis. J. Autoimmun..

[82] Dioguardi M., Crincoli V., Laino L., Alovisi M., Sovereto D., Lo Muzio L., Troiano G. (2020). Prevalence of bacteria of genus actinomyces in persistent extraradicular lesions-systematic review. J. Clin. Med..

[83] Manco M., Putignani L., Bottazzo G.F. (2010). Gut microbiota, lipopolysaccharides, and innate immunity in the pathogenesis of obesity and cardiovascular risk. Endocr. Rev..

[84] Leonardo M.R., Bezerra Da Silva R.A., Assed S., Nelson-Filho P. (2004). Importance of bacterial endotoxin (LPS) in endodontics. J. Appl. Oral Sci..

[85] Herath T.D., Darveau R.P., Seneviratne C.J. (2013). Tetra- and penta-acylated lipid A structures of *Porphyromonas gingivalis* LPS differentially activate TLR4-mediated NF-κB signal transduction cascade and immuno-inflammatory response in human gingival fibroblasts. PLoS ONE.

[86] Day A.E., Langkamp H.H., Bowen L.L., Ascencio F., Agarwal S., Piesco N.P. (1998). Signal transduction during LPS-mediated activation of pulp fibroblasts. J. Dent. Res..

[87] Blix I.J.S., Helgeland K. (1998). LPS from *Actinobacillus actinomycetemcomitans* and production of nitric oxide in murine macrophages J774. Eur. J. Oral Sci..

[88] Matsushita K., Tajima T., Tomita K., Takada H., Nagaoka S., Torii M. (1999). Inflammatory cytokine production and specific antibody responses to lipopolysaccharide from endodontopathic black-pigmented bacteria in patients with multilesional periapical periodontitis. J. Endod..

[89] Jiménez-Beato G., Machuca G. (2005). Heart and periodontal diseases: Does evidence exist of association?. Med. Oral Patol. Oral Cir. Bucal..

[90] Fujita M., Kuraji R., Ito H., Hashimoto S., Toen T., Fukada T., Numabe Y. (2018). Histological effects and pharmacokinetics of lipopolysaccharide derived from *Porphyromonas gingivalis* on rat maxilla and liver concerning with progression into non-alcoholic steatohepatitis. J. Periodontol..

[91] Zhang J., Yu C., Zhang X., Chen H., Dong J., Lu W., Song Z., Zhou W. (2018). *Porphyromonas gingivalis* lipopolysaccharide induces cognitive dysfunction, mediated by neuronal inflammation via activation of the TLR4 signaling pathway in C57BL/6 mice. J. Neuroinflammation.

[92] Kuraji R., Ito H., Fujita M., Ishiguro H., Hashimoto S., Numabe Y. (2016). *Porphyromonas gingivalis* induced periodontitis exacerbates progression of non-alcoholic steatohepatitis in rats. Clin. Exp. Dent. Res..

[93] Kuraji R., Fujita M., Ito H., Hashimoto S., Numabe Y. (2018). Effects of experimental periodontitis on the metabolic system in rats with diet-induced obesity (DIO): An analysis of serum biochemical parameters. Odontology.

[94] Isogai H., Isogai E., Fujii N., Oguma K., Kagota W., Takano K. (1988). Histological changes and some in vitro biological activities induced by lipopolysaccharide from *Bacteroides gingivalis*. Zent. Bakteriol. Mikrobiol. Hyg. A.

[95] Cani P.D., Amar J., Iglesias M.A., Poggi M., Knauf C., Bastelica D., Neyrinck A.M., Fava F., Tuohy K.M., Chabo C. (2007). Metabolic endotoxemia initiates obesity and insulin resistance. Diabetes.

[96] Manco M. (2009). Endotoxin as a missed link among all the metabolic abnormalities in the metabolic syndrome. Atherosclerosis.

[97] Genco R.J., Grossi S.G., Ho A., Nishimura F., Murayama Y. (2005). A proposed model linking inflammation to obesity, diabetes, and periodontal infections. J. Periodontol..

[98] Linden G., Patterson C., Evans A., Kee F. (2007). Obesity and periodontitis in 60-70-year-old men. J. Clin. Periodontol..

[99] Ekuni D., Yamamoto T., Koyama R., Tsuneishi M., Naito K., Tobe K. (2008). Relationship between body mass index and periodontitis in young Japanese adults. J. Periodontal. Res..

[100] Haffajee A.D., Socransky S.S. (2009). Relation of body mass index, periodontitis and Tannerella forsythia. J. Clin. Periodontol..

[101] Lundin M., Yucel-Lindberg T., Dahllof G., Marcus C., Modeer T. (2004). Correlation between TNFalpha in gingival crevicular fluid and body mass index in obese subjects. Acta Odontol. Scand..

[102] Khanna S., Mali A.M. (2010). Evaluation of tumor necrosis factor-alpha (TNF-alpha) levels in plasma and their correlation with periodontal status in obese and non-obese subjects. J. Indian. Soc. Periodontol..

[103] Fentoğlu O., Oz G., Taşdelen P., Uskun E., Aykaç Y., Bozkurt F.Y. (2009). Periodontal status in subjects with hyperlipidemia. J. Periodontol..

[104] Mesia R., Gholami F., Huang H., Clare-Salzler M., Aukhil I., Wallet S.M., Shaddox L.M. (2016). Systemic inflammatory responses in patients with type 2 diabetes with chronic periodontitis. BMJ Open Diabetes Res. Care.

[105] Poole S., Singhrao S.K., Kesavalu L., Curtis M.A., Crean S. (2013). Determining the presence of periodontopathic virulence factors in short-term postmortem Alzheimer’s disease brain tissue. J. Alzheimers Dis..

[106] Kamer A.R., Craig R.G., Dasanayake A.P., Brys M., GlodzikSobanska L., de Leon M.J. (2008). Inflammation and Alzheimer’s disease: Possible role of periodontal diseases. Alzheimer’s Dement..

[107] Qiu C., Yuan Z., He Z., Chen H., Liao Y., Li S., Zhou W., Song Z. (2021). Lipopolysaccharide preparation derived from porphyromonas gingivalis induces a weaker immuno-inflammatory response in BV-2 microglial cells than *Escherichia coli* by differentially activating TLR2/4-mediated NF-κB/STAT3 signaling pathways. Front. Cell Infect. Microbiol..

[108] Hayashi K., Hasegawa Y., Takemoto Y., Cao C., Takeya H., Komohara Y., Mukasa A., Kim-Mitsuyama S. (2019). Continuous intracerebroventricular injection of *Porphyromonas gingivalis* lipopolysaccharide induces systemic organ dysfunction in a mouse model of Alzheimer’s disease. Exp. Gerontol..

[109] Costa M.J.F., de Araújo I.D.T., da Rocha Alves L., da Silva R.L., Dos Santos Calderon P., Borges B.C.D., de Aquino Martins A.R.L., de Vasconcelos Gurgel B.C., Lins R.D.A.U. (2021). Relationship of *Porphyromonas gingivalis* and Alzheimer’s disease: A systematic review of pre-clinical studies. Clin. Oral Investig..

[110] Wang L., Yang Y., Xiong X., Yu T., Wang X., Meng W., Wang H., Luo G., Ge L. (2018). Oral lichen-planus-associated fibroblasts acquire myofibroblast characteristics and secrete pro-inflammatory cytokines in response to *Porphyromonas gingivalis* lipopolysaccharide stimulation. BMC Oral Health.

[111] Chiang C.P., Yu-Fong Chang J., Wang Y.P., Wu Y.H., Lu S.Y., Sun A. (2018). Oral lichen planus—Differential diagnoses, serum autoantibodies, hematinic deficiencies, and management. J. Formos. Med. Assoc..

[112] Zeng Q., Yang X., Chen X., Xia J., Cheng B., Tao X. (2018). *Porphyromonas gingivalis* lipopolysaccharide induces over production of CC chemokine ligand 2 via toll-like receptor-4 in oral lichen planus. J. Oral Pathol. Med..

[113] Lanki M.A., Seppänen H.E., Mustonen H.K., Böckelman C., Juuti A.T., Hagström J.K., Haglund C.H. (2018). Toll-like receptor 2 and Toll-like receptor 4 predict favorable prognosis in local pancreatic cancer. Tumor Biol..

[114] Woo B.H., Kim D.J., Choi J.I., Kim S.J., Park B.S., Song J.M., Lee J.H., Park H.R. (2017). Oral cancer cells sustainedly infected with *Porphyromonas gingivalis* exhibit resistance to Taxol and have higher metastatic potential. Oncotarget.

[115] Scher J.U., Abramson S.B. (2011). The microbiome and rheumatoid arthritis. Nat. Rev. Rheumatol..

[116] Kaur S., White S., Bartold P.M. (2013). Periodontal disease and rheumatoid arthritis: A systematic review. J. Dent. Res..

[117] Arimatsu K., Yamada H., Miyazawa H., Minagawa T., Nakajima M., Ryder M.I., Gotoh K., Motooka D., Nakamura S., Lida T. (2014). Oral pathobiont induces systemic inflammation and metabolic changes associated with alteration of gut microbiota. Sci. Rep..

[118] Klukowska M., Haught J.C., Xie S., Circello B., Tansky C.S., Khambe D., Huggins T., White D.J. (2017). Clinical effects of stabilized stannous fluoride dentifrice in reducing plaque microbial virulence I: Microbiological and receptor cell findings. J. Clin. Dent..

[119] Culshaw S., McInnes I.B., Liew F.Y. (2011). What can the periodontal community learn from the pathophysiology of rheumatoid arthritis?. J. Clin. Periodontol..

[120] Madianos P.N., Bobetsis Y.A., Kinane D.F. (2005). Generation of inflammatory stimuli: How bacteria set up inflammatory responses in the gingiva. J. Clin. Periodontol..

[121] Takeda K., Akira S. (2005). Toll-like receptors in innate immunity. Int. Immunol..

[122] Kinane D.F., Galicia J.C., Gorr S.U., Stathopoulou P.G., Benakanakere M.P. (2008). *gingivalis* interactions with epithelial cells. Front. Biosci..

[123] Hans M., Hans V.M. (2011). Toll-like receptors and their dual role in periodontitis: A review. J. Oral Sci..

[124] Paster B.J., Bches S.K., Galvin J.L., Ericson R.E., Lau C.N., Levanos V.A. (2001). Bacterial diversity in human subgingival plaque. J. Bacteriol..

[125] Xia T., Baumgartner J.C. (2003). Occurrence of actinomyces in infections of endodontic origin. J. Endod..

[126] Schipmann S., Metzler P., Rössle M., Zemann W., von Jackowski J., Obwegeser J.A., Grätz K.W., Jacobsen C. (2013). Osteopathology associated with bone resorption inhibitors—which role does Actinomyces play? A presentation of 51 cases with systematic review of the literature. J. Oral Pathol..

[127] Könönen E., Wade W.G. (2015). Actinomyces and related organisms in human infections. Clin. Microbiol. Rev..

[128] Zijnge V., van Leeuwen M.B.M., Degener J.E., Abbas F., Thurnheer T., Gmür R., Harmsen H.J.M. (2010). Oral biofilm architecture on natural teeth. PLoS ONE.

[129] Kostic A.D., Chun E., Robertson L., Glickman J.N., Gallini C.A., Michaud M., Clancy T.E., Chung D.C., Lochhead P., Hold G.L. (2013). *Fusobacterium nucleatum* potentiates intestinal tumorigenesis and modulates the tumor-immune microenvironment. Cell Host Microbe.

[130] Lee S.A., Liu F., Riordan S.M., Lee C.S., Zhang L. (2019). Global Investigations of *Fusobacterium nucleatum* in human colorectal cancer. Front. Oncol..

[131] Liu L., Liang L., Liang H., Wang M., Lu B., Xue M., Deng J., Chen Y. (2019). *Fusobacterium nucleatum* aggravates the progression of colitis by regulating M1 macrophage polarization via AKT2 pathway. Front. Immunol..

[132] Han Y.W. (2015). *Fusobacterium nucleatum*: A commensal-turned pathogen. Curr. Opin. Microbiol..

[133] Vinogradov E., St Michael F., Homma K., Sharma A., Cox A.D. (2017). Structure of the LPS O-chain from *Fusobacterium nucleatum* strain 10953, containing sialic acid. Carbohydr. Res..

[134] Polak D., Wilensky A., Shapira L., Halabi A., Goldstein D., Weiss E.I., Houri-Haddad Y. (2009). Mouse model of experimental periodontitis induced by *Porphyromonas gingivalis*/*Fusobacterium nucleatum* infection: Bone loss and host response. J. Clin. Periodontol..

[135] Sun Y., Shu R., Li C.L., Zhang M.Z. (2010). Gram-negative periodontal bacteria induce the activation of Toll-like receptors 2 and 4, and cytokine production in human periodontal ligament cells. J. Periodontol..

[136] Jia Y.P., Wang K., Zhang Z.J. (2017). TLR2/TLR4 activation induces Tregs and suppresses intestinal inflammation caused by *Fusobacterium nucleatum* in vivo. PLoS ONE.

[137] Ebbers M., Lübcke P.M., Volzke J., Kriebel K., Hieke C., Engelmann R., Lang H., Kreikemeyer B., Müller-Hilke B. (2018). Interplay between *P. gingivalis*, *F. nucleatum* and *A. actinomycetemcomitans* in murine alveolar bone loss, arthritis onset and progression. Sci. Rep..

[138] Henderson B., Wilson M., Sharp L., Ward J.M. (2002). *Actinobacillus actinomycetemcomitans*. J. Med. Microbiol..

[139] Park C.H., Abramson Z.R., Taba M., Jin Q., Chang J., Kreider J.M., Goldstein S.A., Giannobile W.V. (2007). Three-dimensional micro-computed tomographic imaging of alveolar bone in experimental bone loss or repair. J. Periodontol..

[140] Rogers J.E., Li F., Coatney D.D., Rossa C., Bronson P., Krieder J.M., Giannobile W.V., Kirkwood K.L. (2007). *Actinobacillus actinomycetemcomitans* lipopolysaccharide-mediated experimental bone loss model for aggressive periodontitis. J. Periodontol..

[141] Kozarov E.V., Dorn B.R., Shelburne C.E., Dunn W.A., Progulske-Fox A. (2005). Human atherosclerotic plaque contains viable invasive *Actinobacillus actinomycetemcomitans* and *Porphyromonas gingivalis*. Arter. Thromb. Vasc. Biol..

[142] Aquino-Martinez R., Hernández-Vigueras S. (2021). Severe COVID-19 lung infection in older people and periodontitis. J. Clin. Med..

[143] Marinho V.C.C., Higgins J.P., Logan S., Sheiham A. (2003). Topical fluoride (toothpastes, mouthrinses, gels or varnishes) for preventing dental caries in children and adolescents. Cochrane Database Syst. Rev..

[144] O’Mullane D.M., Baez R.J., Jones S., Lennon M.A., Petersen P.E., Rugg-Gunn A.J., Whelton H., Whitford G.M. (2016). Fluoride and Oral Health. Community Dent. Health.

[145] Friesen L.R., Goyal C.R., Qaqish J.G., He T., Eusebio R., Zsiska M., Farmer T., Schneiderman E. (2017). Comparative antiplaque effect of two antimicrobial dentifrices: Laboratory and clinical evaluations. J. Clin. Dent..

[146] Abdollahi A., Jalalian E. (2019). Effectiveness of Two desensitizer materials, potassium nitrate and fluoride varnish in relieving hypersensitivity after crown preparation. J. Contemp. Dent. Pract..

[147] Haraszthy V.I., Raylae C.C., Sreenivasan P.K. (2019). Antimicrobial effects of a stannous fluoride toothpaste in distinct oral microenvironments. J. Am. Dent. Assoc..

[148] Tasios T., Papageorgiou S.N., Papadopoulos M.A., Tsapas A., Haidich A.B. (2019). Prevention of orthodontic enamel demineralization: A systematic review with meta-analyses. Orthod. Craniofac. Res..

[149] Konradsson K., Lingström P., Emilson C.G., Johannsen G., Ramberg P., Johannsen A. (2020). Stabilized stannous fluoride dentifrice in relation to dental caries, dental erosion and dentin hypersensitivity: A systematic review. Am. J. Dent..

[150] Madléna M., Bánóczy J., Götz G., Márton S., Kaán M., Nagy G. (2012). Effects of amine and stannous fluorides on plaque accumulation and gingival health in orthodontic patients treated with fixed appliances: A pilot study. Oral Health Dent. Manag..

[151] Koopman J.E., van der Kaaij N.C., Buijs M.J., Elyassi Y., van der Veen M.H., Crielaard W., Ten Cate J.M., Zaura E. (2015). The effect of fixed orthodontic appliances and fluoride mouthwash on the oral microbiome of adolescents—A randomized controlled clinical trial. PLoS ONE.

[152] Haught J.C., Xie S., Circello B., Tansky C.S., Khambe D., Sun Y., Lin Y., Sreekrishna K., Klukowska M., Huggins T. (2016). Lipopolysaccharide and lipoteichoic acid binding by antimicrobials used in oral care formulations. Am. J. Dent..

[153] Haught C., Xie S., Circello B., Tansky C.S., Khambe D., Klukowska M., Huggins T., White D.J. (2016). Lipopolysaccharide and lipoteichoic acid virulence deactivation by stannous fluoride. J. Clin. Dent..

[154] Xie S., Haught J.C., Tansky C.S., Klukowska M., Hu P., Ramsey D.L., Circello B., Huggins T.G., White D.J. (2018). Clinical effects of stannous fluoride dentifrice in reducing plaque microbial virulence III: Lipopolysaccharide and TLR2 reporter cell gene activation. Am. J. Dent..

[155] Shaddox L.M., Gonçalves P.F., Vovk A., Allin N., Huang H., Hou W., Aukhil I., Wallet S.M. (2013). LPS-induced inflammatory response after therapy of aggressive periodontitis. J. Dent. Res..

[156] Allin N., Cruz-Almeida Y., Velsko I., Vovk A., Hovemcamp N., Harrison P., Huang H., Aukhil I., Wallet S.M., Shaddox L.M. (2016). Inflammatory response influences treatment of localized aggressive periodontitis. J. Dent. Res..

[157] Nahid M.A., Satoh M., Chan E.K. (2011). MicroRNA in TLR signaling and endotoxin tolerance. Cell Mol. Immunol..

[158] Meilhac O., Tanaka S., Couret D. (2020). High-density lipoproteins are bug scavengers. Biomolecules.

[159] Vesy C.J., Kitchens R.L., Wolfbauer G., Albers J.J., Munford R.S. (2000). Lipopolysaccharide-binding protein and phospholipid transfer protein release lipopolysaccharides from gram-negative bacterial membranes. Infect. Immun..

[160] Brandenburg K., Jürgens G., Andrä J., Lindner B., Koch M.H.J., Blume A., Garidel P. (2002). Biophysical characterization of the interaction of high-density lipoprotein (HDL) with endotoxins. Eur. J. Biochem..

